# A Mechanism for Reliable Mobility Management for Internet of Things Using CoAP

**DOI:** 10.3390/s17010136

**Published:** 2017-01-12

**Authors:** Seung-Man Chun, Jong-Tae Park

**Affiliations:** 1 Gyeongbuk Institute of IT Convergence Industry Technology, Gyeongbuk 38463, Korea; smchun@gitc.or.kr; 2 School of Electronics Engineering, Kyungpook National University, Daegu 41566, Korea

**Keywords:** reliable mobility management, IoT networks, CoAP

## Abstract

Under unreliable constrained wireless networks for Internet of Things (IoT) environments, the loss of the signaling message may frequently occur. Mobile Internet Protocol version 6 (MIPv6) and its variants do not consider this situation. Consequently, as a constrained device moves around different wireless networks, its Internet Protocol (IP) connectivity may be frequently disrupted and power can be drained rapidly. This can result in the loss of important sensing data or a large delay for time-critical IoT services such as healthcare monitoring and disaster management. This paper presents a reliable mobility management mechanism in Internet of Things environments with lossy low-power constrained device and network characteristics. The idea is to use the Internet Engineering Task Force (IETF) Constrained Application Protocol (CoAP) retransmission mechanism to achieve both reliability and simplicity for reliable IoT mobility management. Detailed architecture, algorithms, and message extensions for reliable mobility management are presented. Finally, performance is evaluated using both mathematical analysis and simulation.

## 1. Introduction

In recent years, the Internet of Things (IoT) technology is being increasingly applied to diverse application areas including healthcare monitoring, disaster management, and vehicular management. IoT has been playing an essential role ever since it appeared, which covers from traditional equipment to general household objects [1] and has been attracting the attention of researchers from academia, industry, and government in recent years. The Internet of Things (IoT) is an information network of physical objects (sensors, machines, cars, buildings, and other items) that allows interaction and cooperation of these objects to reach common goals [2]. The IoT will play a key role for building cyber-physical systems for complex industrial applications [3].

Traditional wireless sensor networks are usually being developed using static sensor nodes. However, the applications in IoT environments may require mobility of their nodes for maintaining the Internet connectivity without any need of gateways [4]. There have been proposed lots of Internet Protocol (IP) mobility management protocols, including those standardized by the Internet Engineering Task Force (IETF) such as Mobile IP version 6 (MIPv6) [5] and its variants such as Hierarchical MIPv6 (HMIPv6) [6] and Proxy MIPv6 (PMIPv6) [7]. Unfortunately, most standard IP mobility management protocols may be unsuitable for IoT environments [8]. This is because IoT environments are often constructed with unreliable wireless sensor networks in which hundreds of mobile sensor nodes may be connected to each other. Furthermore, these mobile sensor nodes typically have constrained characteristics in processing capability and electric power.

On the other hand, most standard mobility management protocols such as Mobile IPv6 and its variants typically have high signaling overhead owing to tunneling and binding operations and are considerably complex, thereby incurring high processing overhead [8]. The signaling and processing overhead can result in excessive power consumption during movement of power-constrained IoT sensor nodes. Furthermore, these standard mobility management protocols do not address the characteristics of a constrained wireless network, such as a limitation in packet size, high packet loss ratio, and sleep mode operation. Under unreliable constrained wireless networks for IoT environments, the loss of the signaling message may frequently occur [9]. MIPv6 and its variants do not consider this situation. Consequently, as a constrained device moves around different wireless networks, its IP connectivity may be frequently disrupted and power can be drained rapidly. This can result in the loss of important sensing data or the large delay for time-critical IoT services such as healthcare monitoring and disaster management.

Ahmad et al. [10] recently proposed a power-aware mobility management scheme for IoT communication. However, their work focused on the optimal selection of an access point for the next available network by considering the energy consumption of a particular application during handover. They did not consider the high energy consumption due to signaling and processing overhead during the handover operation. Islam and Huh [11] propose a network-based mobility protocol for an IP sensor network. However, their work focuses on reducing the signaling cost, without considering the latency for the time-critical services such as healthcare monitoring and disaster recovery. Victor et al. [12] proposed the use of a dual-overlay network structure for IP mobility. Their works introduce a second overlay network where the identity-to-location association is stored, but the introduction of a second overlay network structure may lead to an increase in binding update cost as the number of sensor nodes increases.

Ganz et al. [13] presented a resource mobility scheme for service continuity in an IoT environment. They proposed a resource mobility scheme using two operating modes, caching and tunneling, to enable applications to access the sensory data when a resource becomes temporarily unavailable. The sensor gateway caches the measured data, and transmits the data in response to a service provider’s request instead of the sensor. The tunneling method reduces the amount of packet loss during the handover of a sensor by creating a tunnel between the sensor gateways. However, as both sensor gateway and sensor itself can move between different wireless networks, the connectivity might be disrupted during their movement. In summary, most standard mobility management mechanisms based on MIPv6 and its variants may not be suitable for supporting the mobility of mobile sensor nodes in IoT environments because the sensor nodes in such an environment generally have constrained CPU processing power and constrained memory capacities. The sensor nodes usually have a sleep mode operation and are often connected to a unreliable wireless sensor network. Most standard mobility management methods have not addressed these constraints of sensors nodes, and do not consider packet retransmissions during handovers because the IP protocol does not support packet retransmission.

In order to solve the problem mentioned above, in reference [14], we have presented the Constrained Application Protocol (CoAP)-based Mobility Management Protocol (CoMP), which can provide efficient mobility management for constrained mobile sensor nodes in IoT environments. In [14], instead of using the IP protocol, we have used the IETF CoAP [15] for the IoT mobility management. The IETF CoAP is a specialized web transfer protocol for use with constrained nodes and constrained networks in the IoT environment. The protocol is designed for machine-to-machine (M2M) applications such as smart energy and building automation. The CoAP provides a request/response interaction model between application endpoints, supports built-in discovery of services and resources, and includes key concepts of the Web such as Uniform Resource Identifiers (URIs) and Internet media types. The integration of devices with contents on the Web can be easily done by the use of CoAP because it takes into account requirements such as low signaling overhead, multicast support, and simplified architecture for constrained environments with limited resources. Therefore, the requirements offered by CoAP—low signaling overhead, multicast support, and simplified architecture—suits constrained devices with limited processing capacity, low power, low memory, and poor Internet connectivity.

In this paper, we have proposed a reliable mobility management mechanism for IoT using the IETF CoAP protocol. We have extended the previous work, which was published in [14]. More specifically, we have presented the detailed algorithm of the reliability mechanism for IoT mobility management. Then, we have mathematically derived the performance measures of the reliability mechanism, such as handover delay. In deriving the performance measures, we have taken into account the IoT infrastructure characteristic of packet fragmentation, which is caused by the limited packet size of the IEEE 802.15.4 low data rate personal area network. Based on this mathematical analysis, the performance of the reliability mechanism has been evaluated by simulation. It is found that the simulation results are consistent with the mathematical results.

As mentioned before, there have been lots of IP mobility management protocols proposed such as MIPv6 and its variants, which are standardized by IETF [16]. However, none of these standard mobility management protocols provide any reliability mechanisms. Compared with our previous work in [14], the contributions of our research here are summarized as follows:

- The procedure of the reliable mobility management using CoAP have been presented for the IoT environment. More specifically, a detailed signaling procedure for achieving reliable mobility management has been designed.
- A detailed algorithm has been designed for implementing the reliability mechanism of CoAP-based mobility management. In particular, the retransmission mechanism of CoAP has been utilized to implement the reliability mechanism. This greatly simplifies the design of the efficient reliability mechanism. The confirmable (CON) message of the CoAP protocol is extended for reliable transmission of the signaling message under an unreliable IoT network environment.
- We have mathematically derived the performance measures of the reliability mechanism such as average handover delay and total handover delay. In deriving the performance measures, we have taken into account the packet fragmentation effect, which is caused by the constrained packet size of the IEEE 802.15.4 low data rate wireless network. Based on this mathematical analysis, the performance of the reliability mechanism has been evaluated by simulation. It is found that the simulation results are consistent with the mathematical results.

The remainder of this paper is organized as follows. In Section 2, the architecture and procedure of reliable mobility management are described. In Section 3, the detailed algorithm for reliable mobility management is presented. Mathematical analysis for the performance of this algorithm is also presented. In Section 4, we describe the performance results of the proposed reliability mechanism for IoT mobility management. Finally, in Section 5, we provide some concluding remarks regarding this research.

## 2. Architecture and Procedure of Reliable Mobility Management

In this section, reliable mobility management architecture is described. We also describe the detailed signaling procedure for reliable mobility management.

### 2.1. Architecture for Reliable Mobility Management

**Figure 1:**
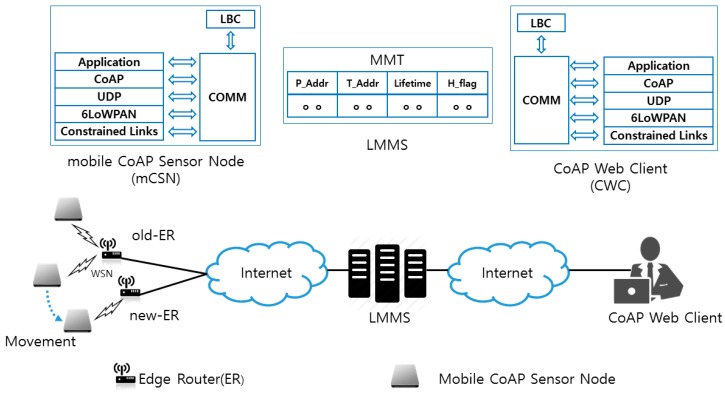
Reliable mobility management architecture using Constrained Application Protocol (CoAP)-based Mobility Management Protocol CoMP.

In Figure 1, we present the reliable mobility management architecture using CoAP-based Mobility Management Protocol (CoMP). The components of the architecture consist of a mobile constrained sensor node (mCSN), IoT Mobility Management Server (IMMS), and CoAP Web Client (CWC). The IMMS maintains the location information in the Mobility Management Table (MMT), which is necessary to perform mobility management, thereby managing the changed IP address of an mCSN. The CWC is a Web browser equipped with the CoAP protocol. The protocol stack of the mCSN consists of IEEE 802.15.4 Medium Access Control (MAC), IPv6 over Low Power Personal Area Network (6LoWPAN), CoAP, and CoMP. A local binding cache (LBC) locally maintains the permanent IP address P_Addr, temporary IP address T_Addr, and Lifetime, which is retrieved by the edge router in the visited network by accessing the Dynamic Host Configuration Protocol (DHCP) server.

In the following, we present the CoMP handover procedure, which consists of four procedures—registration, connection, binding, and hold—to provide the mobility management. The signaling messages of CoMP use the Confirmable (CON) message of CoAP. First, the mCSN and CWC register their own P_Addr and T_Addr at the MMT of the IMMS by exchanging CoAP POST request/response messages. As the CWC attempts to establish a connection with the mCSN, the CWC sends a GET request message to the IMMS to retrieve the IP addresses of an mCSN. As a response, the CWC receives an Acknowledge (ACK) response message with the current T_Addr and its Lifetime, which are stored at the LBC of the mCSN. Then, the CWC stores the T_Addr and Lifetime in the LBC of the CWC. Afterwards, the CWC can exchange data with the mCSN directly.

Now, let us consider the case where the mCSN moves from an old edge router (old-ER) to a new edge router (new-ER). As the mCSN moves away from the old-ER and enters into the network domain of the new-ER, it requires an IP handover operation. To accomplish this, the mCSN first detects the radio signal strength (RSS) from the access point or base station connected to the old-ER. As the RSS from the old-ER drops below a threshold value, the mCSN prepares the handover operation in advance.

Before the mCSN performs the handover, it notifies the handover start operation by exchanging a Hold message with the IMMS. The Hold message uses the CoAP PUT request/response message with a Hold flag (H_Flag). H_Flag indicates that the mCSN is performing the handover procedure. The mCSN cannot receive the request of the CWC until H_Flag is set to “0” by the PUT request message for binding update. Thus, the usage of H_Flag can prevent packet loss during the handover operation of the mCSN. The PUT messages can also be used for status notification as the mCSN experiences a power failure or enters into the sleep mode.

As the mCSN moves into the overlapped region of the two network domains which are covered by old-ER and new-ER, the mCSN attempts to acquire a new temporary IP address (i.e., T_Addr) from the new-ER through DHCP. Then, the mCSN sends a PUT request message for binding update to the IMMS. The message includes T_Addr, P_Addr, and the H_Flag, which is set to “0”. As the IMMS receives the PUT request message, it sends it to the CWC by referring to MMT. As the IMMS and CWC receive the PUT request message for binding update, they update the T_Addr and H_Flag in the MMT and LBC. The IMMS and CWC can then retrieve the data from the mCSN in the new network domain. In this way, the CWC and the mCSN can exchange the data without packet loss during the handover operation. The CoMP signaling messages are configured by extending the option delta and payload in the CoAP message format [17].

### 2.2. Procedure for Reliable Mobility Management

**Figure 2:**
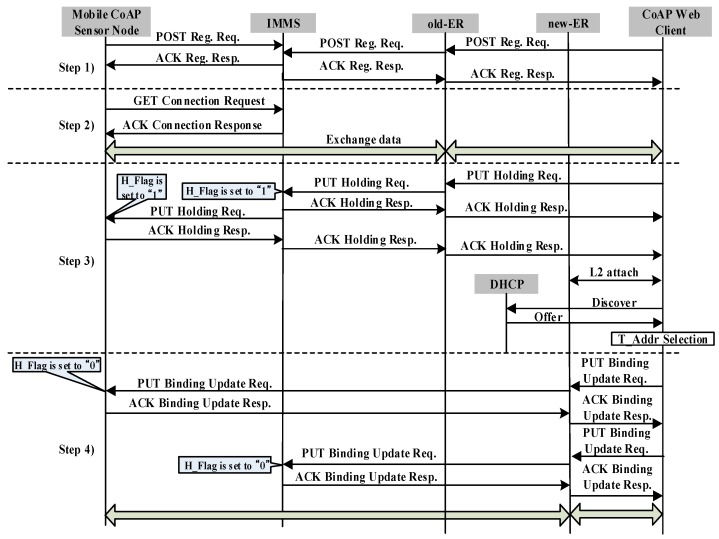
Signaling flow diagram of CoMP.

Figure 2 shows the signaling flow diagram for CoMP. All control messages use a confirmable message as Figure 2 indicates. The signaling flow diagram includes registration, connection, holding, and binding. We assume that mCSN obtains the P_Addr, T_Addr, and Lifetime from old-ER.

Step 1: Both CWC and mCSN register their own P_Addr and Lifetime in the MMT of the IMMS by exchanging a POST registration request message and ACK registration response message.

Step 2: CWC performs the connection procedure to retrieve the destination IP address of the T_Addr and the P_Addr of mCSN. To accomplish this, CWC sends a GET connection request message to the IMMS. As a response, CWC receives an ACK connection response, which includes the T_Addr and Lifetime of mCSN in the MMT of the IMMS. CWC then inserts the T_Addr and Lifetime with the corresponding P_Addr of mCSN in the LBC. CWC then attempts to connect to mCSN by referring to P_Addr and T_Addr. Subsequently, CWC can exchange data with mCSN directly until the Lifetime of T_Addr of mCSN expires.

Step 3: We consider the case where mCSN moves from the network domain covered by old-ER to that of the new-ER. As mCSN moves away from old-ER and enters the network domain of new-ER, it requires an IP handover operation. To perform the handover operation, mCSN first detects the radio signal strength (RSS) from the access point or base station connected to the old-ER. As mCSN detects a degradation of the RSS from the access point or base station connected to the old-ER, it sends a PUT holding request message to the IMMS to hold the request messages from the other CoAP nodes to prevent packet loss of the request message of other CoAP nodes. As the IMMS receives the PUT holding request message, the IMMS updates the H_Flag of mCSN in the MMT to “1”. IMMS then sends the PUT holding request message to CWC. Next, CWC updates the H_Flag in the LBC to “1”. As a response, CWC sends an ACK holding response message to the IMMS.

Because the RSS from the access point or base station connected to the old-ER is less than the threshold value, mCSN cannot send or receive data from old-ER, and disconnects a connection from the access point or base station connected to the old-ER. mCSN then discovers the new-ER. mCSN attempts to perform the connection attachment to the access point or base station connected to the new-ER. It then retrieves a new T_Addr through the DHCP server, which includes both Discovery and Offer procedures.

Step 4: After obtaining a new T_Addr, mCSN sends a PUT binding update request message to both the IMMS and CWC through new-ER, simultaneously, by referring to the T_Addr of CWC in the LBC, to inform them of the new T_Addr. The PUT binding update (BU) request message includes the P_Addr, T_Addr, and Lifetime of mCSN. After the IMMS and mCSN receive the PUT BU message, the T_Addr and Lifetime are updated in the MTT and LBC and the H_Flag is set to “0”. Finally, CoMP node A can retrieve the data from mCSN. In this manner, the connection between CWC and mCSN can be seamlessly maintained during the handover operation.

## 3. Reliable Message Transmission Algorithm and Analysis of Handover Delay of CoMP

In this section, we describe the algorithm of signaling message retransmission for a reliable mobility management algorithm of CoAP-based Mobility Management Protocol (CoMP), and analyze the performance of the handover latency. The CoMP extends the CON message of the CoAP protocol to reliably transmit the signaling message under an unreliable IoT network environment. In particular, the retransmission is instrumented by using a Message ID and CON message of the CoAP protocol, using CoAP PUT message. The Message ID is used to detect duplicates and for transmission reliability, and the CON message is retransmitted using both default timeout and exponential back-off between retransmissions until the recipient sends an ACK with the same Message ID from the corresponding endpoint. Algorithm 1 shows the algorithm for reliable transmission of CoAP message where *T*, *Rc*, and *m* are timeout, retransmission count, and maximum number of retransmission counts, respectively. The algorithm is self-explanatory, and extends the CoAP retransmission algorithm [15]. In case of packet loss, the PUT messages are retransmitted for binding update and holding.

**Algorithm 1:** Algorithm of Reliable CoAP Signaling Message Transmission.Algorithm Reliable_Signaling_Message_Transmission (*P*, *T*, *Rc*, *m*); /* This algorithm describes the reliable transmission of CoAP message by using *T*, *Rc* and *m* */ *P*: CoAP message with sequence number *T*: Timeout//T = ACK_TIMEOUT*ACK_RANDOM_FACTOR [13] *Rc*: Retransmission count//initially *Rc* = 0 *m*: Maximum number of retransmission counts//*m* = 4
1**Begin**2**If** (*T* == 0 OR *Rc* == 0) **Then {**3 ***T*** *=* ACK_TIMEOUT*ACK_RANDOM_FACTOR;4 *Rc* = 0; *m* = 4;}5**Else** **{**6   **If** (*Rc* < *m*) **Then {**7    Send the CoAP message to lower layer;8    **While** (*T < Timeout*)//wait until *T* is expired9       **If** (mCSN receives Acknowledgement)10     **Then** **{***T* = 0; *Rc* = 0;11        **Call** CoAP_Retransmission (*P + 1*, *T*, *Rc*, *m*);}12     **Else** **Call** CoAP_Retransmission (*P*, T*2, *Rc + 1*, *m*);}13    **Else** Discard *P*;} // *P* is discarded14**End**

The packet loss of the signaling message can dramatically increase the handover latency, which may be caused by collision, congestion, and system failure in both wireless and wired communication links. The retransmission of signaling messages in the case of packet loss may greatly reduce the handover latency in constrained IoT networks. To analyze the handover latency of CoMP, we first derive the average number of transmissions and retransmissions considering the CoAP retransmission mechanism. In [17], the probability of CoAP packet error rate by the impact of Signal-to-Noise Ratio (SNR) was investigated for a CoAP message composed of *f* fragmented packets. The author [17] studied the impact of the SNR on the physical level packet loss rate of an 802.15.4 link. By [17], the packet loss rate *P* is given in Equation (1) below:

(1) $$P=1-{\left(1-S\right)}^{2m}$$

where *S* is the symbol error rate and m is the length in bytes of the MAC packet. The symbol error rate *S* is related to the SNR [17].

The probability A of having an erroneous CoAP packet at the application layer, including the retransmission at the MAC layer, is derived as $A=P^{\left(r+1\right)}$, where *r* is the maximum number of retransmissions allowed for the MAC layer (default value is 5). However, the entire IEEE 802.15.4 maximum transmission unit (MTU) is 127 bytes. Hence, the CoAP packet must be fragmented. If a CoAP message is comprised of *f* fragments, the probability of CoAP packet error rate $A_{f}$ is given by:

(2) $$A_{f}=\overset{f}{\underset{j=0}{\sum }}A\times {\left(1-P\right)}^{j-1}$$

where the maximum number of fragments is fixed to 12. In [17], the packet error rate by impact of SNRs (1, 1.5, 2, and 2.5 dB) was investigated.

We derive the average number of retransmission counts and average number of transmissions for a simple stop-and-wait mechanism of CoAP with packet-error rate $A_{f}$. The average number of retransmission counts *R* for a CoAP message with *f* fragmented CoAP messages is derived as:

(3) $$R=\overset{m-1}{\underset{i=0}{\sum }}i\left(1-A_{f}\right)A_{f}^{i}+\left(m-1\right)A_{f}^{m}$$

where *m* is maximum number of retransmissions with a typical value of 4 and *I* is the retransmission count. The average number of transmissions *S*, including retransmission for a CoAP packet, is calculated as:

(4) $$S=\overset{m}{\underset{i=1}{\sum }}i\left(1-A_{f}\right)A_{f}^{i-1}+mA_{f}^{m}$$

With a maximum *m* retransmissions, the probability of a CoAP messages not being transmitted is $A_{f}^{m+1}$. The average retransmission delay *D* for transmitting a CoAP signaling message can be calculated as:

(5) $$D=\sum_{k=0}^{m-1}(1-A_{f})A_{f}^{k}\left\{T_{r}+\left(2^{k}-1\right)T_{w}\right\}+A_{f}^{m}\left(2^{m}-1\right)T_{w}$$

where *k* is the retransmission count and the timeout $T_{w}$ is the amount of time that the mCSN waits for an acknowledgement packet from a remote device. The roundtrip time $T_{r}$ is the time from the start of the transmission until an ACK message is received. It is approximately equal to the end-to-end delay. The derivation of the above Equation (5) is included in the Appendix.

Using the average delay of a CoAP message derived above, we can calculate the total handover latency of CoMP. The handoff latency at the mCSN is the time interval during which an mCSN cannot send or receive any packets during a handover operation. The total handover latency consists of the link switching time ($t_{L2}$), which is caused by the L2 handover between the new-ER and mCSN, movement detection delay ($t_{MD}$) at the mCSN, IP configuration delay and duplicate address detection by the DHCP ($t_{DHCP}$), and location update latency ($t_{BU}$). $t_{BU}$ is the sum of $2t_{\left(mCSN,IMMS\right)}$ and $2t_{\left(IMMS,CWC\right)}$, which are the transmission delay times of the PUT request/response messages for binding update between the mCSN and IMMS and between the IMMS and CWC, respectively. The total handover delay $T_{CoMP}$ is then calculated as follows:

(6) $$T_{CoMP}=t_{L2}+t_{MD}+t_{DHCP}+2t_{\left(CSN,IMMS\right)}+2t_{\left(IMMS,CWC\right)}$$

It is noted that $t_{BU}$ increases as the retransmission count increases.

## 4. Performance Evaluation

Table 1 presents the basic parameters for evaluating the performance of CoMP. The parameters used in this analysis were set to typical values found in [18]. To analyze the average number of transmissions and retransmissions and the handover latency, we used the packet loss rate by SNRs with *f* fragments of a CoAP message in [17]. It is assumed that the wireless link bandwidth and wired link bandwidth were 250 Kbps and 100 Mbps, respectively.

**Figure 3:**
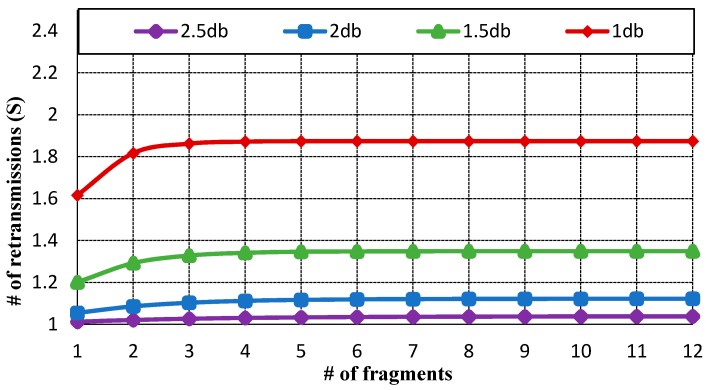
Average number of retransmission counts for *f* fragmented CoAP messages.

The quality of service requirements for the handover delay depends on the message type of the IoT application, such as discrete or continuous message data. Figure 3 displays the average number of retransmissions. As the probability of packet error rate is lower than 30%, the CoAP message is retransmitted only once.

**Figure 4:**
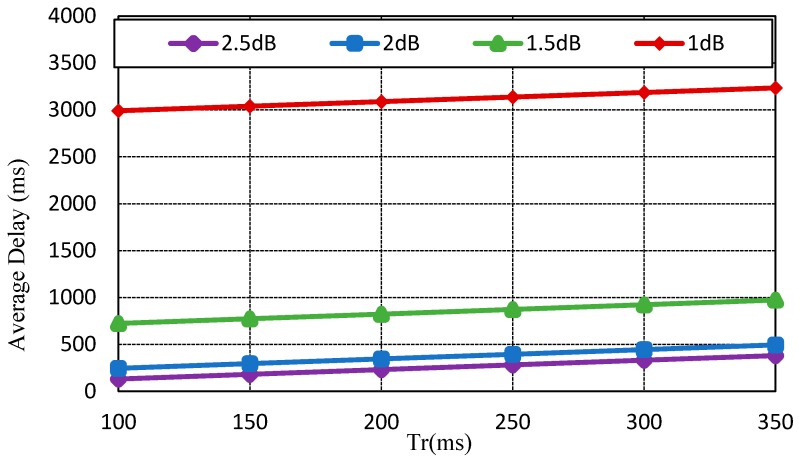
Average delay of signal-to-noise ratios (SNRs) by impact of roundtrip time $T_{r}.$

**Figure 5:**
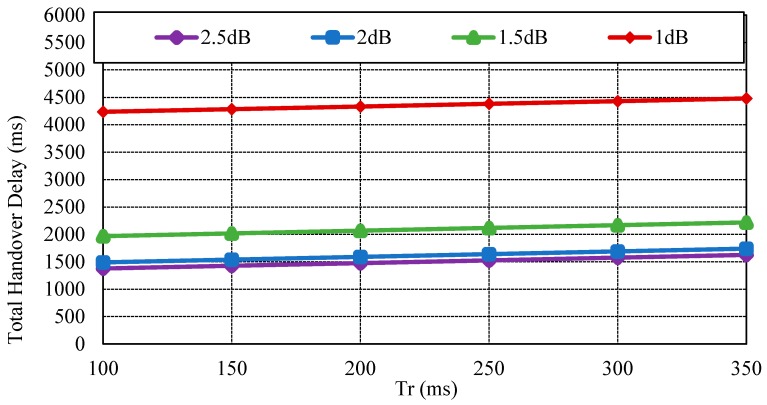
Total handover delay with considering CoAP retransmission mechanism by impact of $T_{r}$.

However, as the probability of packet error rate becomes 50%, the CoAP message is retransmitted approximately twice. Figure 4 indicates the average delay of SNRs by impact of transmission delay ($T_{r}$). As the retransmission count increases, the average delay also increases. If the CoAP message is retransmitted due to packet error or wireless network failure, the average delay is approximately 3 s. Figure 5 exhibits the total handover delay with the CoAP retransmission mechanism by impact of $T_{r}$. As the packet loss rate is approximately 50%, the handover latency increases to 4.3~4.5 s. In this way, CoMP supports reliability in the handover operation. Conversely, the related standard mobility protocols such as MIPv6 and HMIPv6 do not support reliability. As the failure of signaling message occurs, these mobility protocols do not support an automatic retransmission mechanism.

**Figure 6:**
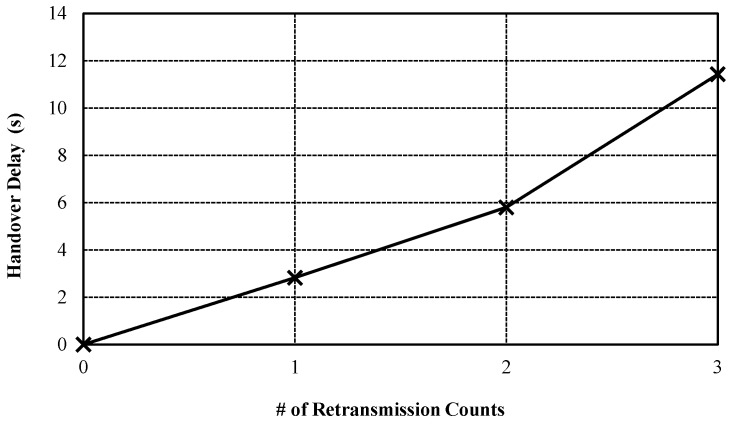
Handover delay by increasing retransmission counts.

Figure 6 shows the handover delay by retransmission counts. Although the handover delay due to the retransmission can be increased, the CoMP can guarantee the reliability of handover message by using CoAP retransmission mechanism. On the contrary, the related standard mobility protocols such as MIPv6 do not support the retransmission mechanism. As result, CoMP can be utilized to a high reliability service such as remote healthcare service on IoT environment.

## 5. Conclusions

In this paper, we have presented a mechanism for reliable IoT mobility management using CoAP. In particular, the detailed algorithm and performance analysis are presented for achieving the reliability of mobility management in Internet of Things environments. Most standard mobility management protocols at the network layer such as MIPv6 and its variants do not consider packet retransmissions during handovers because the network layer does not support packet retransmission. In this paper, we have extended the CON message of the CoAP protocol for reliable transmission of the signaling message under an unreliable IoT network environment. A detailed mathematical analysis of the retransmission model for supporting the reliable mobility management has been presented. Finally, performance is evaluated by both mathematical analysis and simulation. The performance evaluation confirmed that the proposed mechanism can effectively support reliable mobility management. Further study may be required to provide the security aspect of the proposed mechanism.

## Figures and Tables

**Table 1:** Parameters for delay analysis (Kbps, ms, bytes).

Param	Value	Param	Value	Param	Value
*t_DHCP_*	5000	*t*_(*mCSN*, *ER*)_	4.4	*t*_(*CWC*, *IMMS*)_	50
*t_MD_*	100	*t*_(*mCSN*, *IMMS*)_	79.4	*S*	113
*t_L2_*	50	*t*_(*mCSN*, *CWC*)_	79.4	*m*	4

## Appendix A

Derivation of Equation (5): $D=\overset{m-1}{\underset{k=0}{\sum }}(1-A_{f})A_{f}^{k}\left\{T_{r}+\left(2^{k}-1\right)T_{w}\right\}+A_{f}^{m}\left(2^{m}-1\right)T_{w},$ where *D* is the average retransmission delay *D* for transmitting a CoAP signaling message, and *k* is the retransmission count, and the timeout $T_{w}$ is the amount of time that the mCSN waits for an acknowledgement packet from a remote device. The roundtrip time $T_{r}$ is the time from the start of the transmission until an ACK message is received.

Proof: First, we introduce a model for this delay that adopts the CoAP retransmission mechanism, and analyze handover delays based on this model. To estimate the handover delay, we measure the average retransmission delay with the exponential back-off for a confirmable message. Table A1 shows the model for the average retransmission delay of a CoAP message. In Table A1, a white box indicates that the CoAP packet was successfully transmitted to another CoAP node, and a checked box indicates that the CoAP packet was not transmitted to the CoAP node due to the lossy network environment.

The model’s parameters and notations are as follows:

*T_w_*: The timeout, *T_w_*, is the time that the CoAP node waits for an acknowledgement packet from a remote device before resending the last local confirmable message. The initial timeout is set to a random duration (often not an integral number of seconds) between the ACK_TIMEOUT and (ACK_TIMEOUT * ACK_RANDOM_FACTOR). In the CoAP standard [15], the ACK_TIMEOUT and ACK_RANDOM_FACTOR are transmission parameters, with default values of 2 and 1.5 s, respectively. In implementing CoAP, the initial timeout is set to a random value between 2000 and 3000 ms. When *T_w_* is triggered and the retransmission counter is less than the maximum number of retransmissions, the message is retransmitted. The retransmission counter is then incremented, and the next timeout is doubled.

*T_r_*: The roundtrip time, *T_r_*, is the time from the start of the sending node’s transmission to the receipt of the acknowledgement message by the same CoAP node. *T_r_* is the sum of the average back-off time period, the packet-transmission time, the transceiver’s transmitting-to-receiving turnaround time, the ACK transmission time, and the Inter Frame Spacing (IFS) time. Details surrounding the transmission delay for IEEE 802.15.4 can be found in reference [19]. From this reference [19], we set T*_s_* to 4.4 milliseconds in a 60-byte payload to evaluate the performance.

*r*: The retransmission counter, *r*, indicates the number of retransmissions, which increases every time the CoAP node does not receive an acknowledgement message within *T_w_*. In the CoAP standard, the maximum value, *m*, of the retransmission counter is set at 4. The maximum number of retransmissions is therefore 3, because the initial retransmission count is zero.

*A_f_*: *A_f_* is the probability that the CoAP signaling message with *f* fragments will not be successfully transmitted. We discussed this probability in the previous section. Table A1 shows the model for the average delay in transmitting a CoAP message, including the CoAP retransmission mechanism. The CoAP node sends a confirmable message after setting the timeout *T_w_* for sending confirmable messages. Given that the local CoAP node receives the acknowledgement message, the transmission delay is *T_r_*. At that time, the probability of transmission failure for each CoMP signaling message is *A_f_*. However, if the local CoAP node does not receive the acknowledgement message within *T_w_*, the CoAP node attempts its first retransmission of the message after the timeout is set to 2*T_w_* and the retransmission counter is increased. At that time, the probability of the occurrence of a single retransmission is $\;\left(1-A_{f}\right)A_{f}$.

**Table A1:** Model for the average delay in transmitting a CoAP message.

Retransmission. Counter (a)	Probability of Retransmission	Sojourn Time	Transmission Model of a CoAP Signaling Message
0	$\left(1-A_{f}\right)$	$T_{r}$	
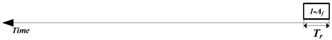
1	$\left(1-A_{f}\right)A_{f}$	$T_{r}+T_{w}$	
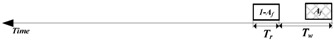
2	$\left(1-A_{f}\right)A_{f}^{2}$	$T_{r}+2T_{w}+T_{w}$	
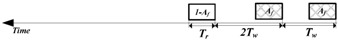
3	$\left(1-A_{f}\right)A_{f}^{3}$	$T_{r}+4T_{w}+2T_{w}$	
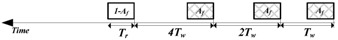
4	$A_{f}^{4}$	$8T_{w}+4T_{w}+2T_{w}$	
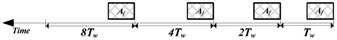

We can conclude that after several retransmission attempts, the average waiting time will converge to *T_r_* + *T_w_* for the first retransmission, *T_r_* + 2*T_w_* for the second retransmission, and *T_r_* + 3*T_w_* for the third retransmission. The retransmission attempts are repeated until the retransmission counter reaches *m*. Because the probability of the occurrence of a first retransmission is $\left(1-A_{f}\right)A_{f}$, the average retransmission delay for a CoAP signaling message on its first retransmission attempt becomes $\left(T_{r}+T_{w}\right)\times \left(1-A_{f}\right)A_{f}$, as shown in Table A1. As shown in Table A1, the average retransmission delay for a single CoAP signaling message on its second retransmission attempt is $\left(T_{r}+T_{w}+2T_{w}\right)\times \left(1-A_{f}\right)A_{f}^{2}$. The average retransmission delay for a CoAP signaling message on its third retransmission attempt is $\left(T_{r}+4T_{w}+2T_{w}+T_{w}\right)\times \left(1-A_{f}\right)A_{f}^{3}$, and the average retransmission delay for a CoAP signaling message on its fourth retransmission attempt is $\left(T_{r}+8T_{w}+4T_{w}+2T_{w}+T_{w}\right)\times A_{f}^{4}$. Therefore, the average retransmission delay, *D*, for transmitting a CoAP signaling message considering the exponential back-off retransmission mechanism can be calculated as follows: $D=\overset{m-1}{\underset{k=0}{\sum }}(1-A_{f})A_{f}^{k}\left\{T_{r}+\left(2^{k}-1\right)T_{w}\right\}+A_{f}^{m}\left(2^{m}-1\right)T_{w}.$
